# Access to and use of contraceptive care during the first COVID-19 lockdown in the UK: a web-based survey

**DOI:** 10.3399/BJGPO.2021.0218

**Published:** 2022-08-10

**Authors:** Richard Ma, Kimberley Foley, Sonia Saxena

**Affiliations:** 1 Child Health Unit, Department of Primary Care and Public Health, Imperial College London, London, UK

**Keywords:** SARS-CoV-2, COVID-19, contraception, general practice, health services research, surveys and questionnaires

## Abstract

**Background:**

The first wave of lockdown measures to control the COVID-19 pandemic in the UK resulted in suspension of ‘non-essential’ services, including contraceptive care.

**Aim:**

To examine women’s perceptions and experiences of contraceptive care in the UK during the first lockdown.

**Design & setting:**

A cross-sectional survey during the lockdown period from March–June 2020.

**Method:**

An online questionnaire was designed asking women aged 16–54 years their experiences of contraceptive care during lockdown. Questions were based on Maxwell’s evaluation framework on access, acceptability, relevance or appropriateness, and equity. It was promoted on social media from 27 May–9 June 2020. A descriptive analysis was conducted of quantitative data and thematic analysis of free-text data.

**Results:**

In total, 214 responses were analysed. General practice was the source of contraception for 43.4% (*n* = 49) and 52.3% (*n* = 34) of responders before and during the lockdown, respectively. The study found 55.1% (*n* = 118) of responders, including regular and new users, were uncertain where or how to get contraception during the pandemic. Responders reported reduced access to contraception during lockdown, and some thought sexual health clinics and general practices were closed. Remote consultations and electronic prescriptions facilitated contraceptive access for some responders. Long-acting reversible contraception (LARC) was unavailable in some areas owing to restrictions, and alternatives were not acceptable to those who used methods for non-contraceptive benefits to treat medical conditions; for example, menorrhagia.

**Conclusion:**

The study highlighted the need for better information and signposting for contraception during lockdown. Contraception, including LARC, should be reframed as an essential service with robust signposting for pandemic planning and beyond.

## How this fits in

Lockdown measures to contain COVID-19 reduced access to routine health services globally, including contraceptive care, which was not regarded as an ‘essential service’ in some countries such as the UK. This is a survey of women’s perceptions and experiences of contraceptive care during the first UK lockdown from March–June 2020. It found over half (55.1%, *n* = 118/214) of responders were unclear about how to access contraception during lockdown as they thought general practices and sexual health clinics were closed. Remote contraceptive consultations were helpful during the lockdown, but women wanted better signposting for contraception and access to LARC. The findings have useful contributions for the planning of future pandemics.

## Introduction

Widespread containment measures, such as national lockdowns, to control the SARS-CoV-2 pandemic resulted in reduced access to routine health services globally, including contraceptive care. In many countries, including the UK, much of primary care, including contraception and long-term conditions management, were deprioritised. A systematic review of over 20 countries reported health care use fell by about one-third during the pandemic.^1,2^

The UK government introduced the first national lockdown measures from 23 March 2020 to deal with the first wave of SARS-CoV-2 infections spreading in the community. Each of the devolved UK nations applied different measures and eased them at different times; the restrictions in England were eased in June 2020.^3–5^ General practices were advised to use total triaging and remote models to assess clinical need for face-to-face consultations in the first wave of lockdown.^6^ The triaging measures resulted in one-third fewer appointments in general practices in April and May 2020 overall compared with the previous year, as more remote consultations were adopted.^7^ These restrictions were relaxed in subsequent lockdowns. Among the advice from the Royal College of General Practitioners and British Medical Association on reprioritisation of clinical services was contraception could continue ‘if possible‘ and provision of LARC, such as injections and implants, should stop.^8^

Before the pandemic, the proportion of conceptions leading to abortions had been increasing, suggesting unmet need and access to contraception were already a problem for women of reproductive age (16–54 years) in the UK.^9^ Further disruption to contraceptive provision would have had adverse impact on unwanted pregnancies, particularly among young and vulnerable groups, not least because general practice had always been a significant provider of contraceptive services.^10,11^ This concern was not unique to the UK as a global survey of health providers, researchers, and policymakers conducted in May 2020 reported women’s health would suffer because of reduced contraceptive provision.^12^

A survey of women’s access to female healthcare services in the UK conducted during the pandemic reported poor signposting about which contraception and sexual health (CASH) clinics were open and what was available in their area.^13^ However, it did not specifically report access to LARC, and responses were limited to structured survey questions. In addition to reduced access to contraception in general, the authors speculated the curtailment of LARC and recommendations to use different methods, such as condoms, led to unmet contraception needs for some women during lockdown. Little is known about the impact of lockdown for women of reproductive age (16–54 years) and their experiences on contraceptive access, especially LARC. The aim of this study, therefore, was to examine the impact of the first wave of COVID-19 lockdown on access and experience of obtaining contraception from providers in the UK, especially primary care. This survey was conducted in a short-time frame to submit to a UK Cross-Party Parliamentary Inquiry into contraceptive care during the pandemic.^14^

## Method

A semi-structured online questionnaire was designed, which included free-text comments to examine contraceptive care experienced by the target population of women aged 16–54 years who were using or seeking contraception during the first COVID-19 pandemic lockdown (see Supplementary Figure S1). Questions were developed on access, acceptability, relevance or appropriateness, and equity (Table 1), based on Maxwell’s dimensions of quality; this framework has been used to evaluate contraceptive services in the past.^15,16^ The following three scenarios were speculated when women would need contraceptive care from providers during the pandemic: women who were not on contraception at the time of the survey but were considering their options; those who obtained contraception before the lockdown and needed a change or repeat prescription; and those who obtained contraception during the lockdown. Using adaptive questioning (where the sequence of questions depended on the answers given), responders were streamed to one of these three mutually exclusive groups according to their contraceptive use at the time of the survey to ensure the questions were relevant.

**Table 1.** Maxwell’s evaluation dimensions and related sources of data from survey

Dimension	Survey questions
Access	‘Where did you get your method of contraception?’’Do you expect any problems getting future supplies?’’Were you able to obtain contraception that you wanted (before/after lockdown)?’
Acceptability	’Did you have any problems getting your supply?’’Were you happy with the alternative method?’
Relevance or appropriateness	Survey question: ’Did you get the method you wanted/needed?’
Equity	Demographic data, for example, age, sex, location, and if this was the usual place of residence during lockdown.

Qualtrics software was used (https://www.qualtrics.com) to create questionnaire materials, and the draft survey was piloted with several members from a patient and public involvement group. After this, feedback changes were made including addition of lactational amenorrhoea method (LAM) and correction of an adaptive question. The depot progestogen contraceptives ‘Depo-Provera‘ and ‘Sayana Press‘ were separated because the latter could be self-administered, so did not require a face-to-face appointment with a healthcare professional. Further information about the survey is in the CHERRIES (Checklist for Reporting Results of Internet E-Surveys) reporting checklist.^17^ No incentives were given for completion, participation was voluntary, and responders could withdraw at any time. The survey was promoted on social media (Twitter, Facebook, and Instagram) (see Supplementary Figures S2–S4). The survey went live for 2 weeks from 27 May 2020–9 June 2020.

Data were analysed using Microsoft Excel and NVivo (version 12) to organise free-text data for thematic analysis. Two authors analysed the free-text data independently, then compared the findings noting any convergence, complementarity, or discrepancy.^18^ The manuscripts were prepared using the following two reporting frameworks: CHERRIES and STROBE (Strengthening the Reporting of Observational Studies in Epidemiology).^17,19^

## Results

### Responder profile

There were 363 visits to the survey site from 352 unique IP addresses between 27 May 2020 and 9 June 2020. A total of 277 responders consented and agreed to participate, 59 did not answer the demographic questions, resulting in a completion rate of 78.7% (*n* = 218/277); four cisgender men were excluded from this sample to make 214 eligible responders (see Supplementary Figure S5).

Responders were aged between 16 and 54 years (see Supplementary Table S1); the largest age group was 16–18 years (32.7%, *n* = 70), smallest was 45–54 years (3.7%, *n* = 8); there were responders from all UK regions with the largest number from South East England (14.5%, *n* = 31) and the smallest from Northern Ireland (2.8%, *n* = 6).

**Figure 1.**
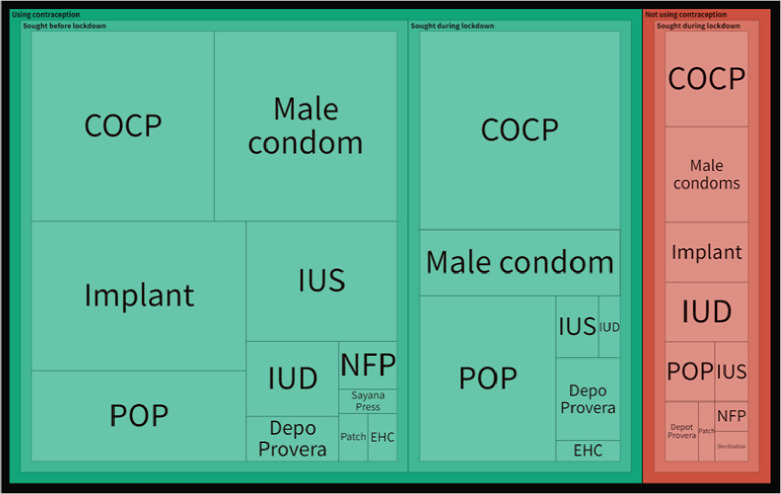
Tree diagram summarising contraception used or sought by responders at the time of survey. Size of each box corresponds to the number of women using or seeking that method. Long-acting reversible contraceptive (LARC) methods include: implant, IUD, IUS, Depo-Provera, and Sayana Press. COCP = combined oral contraceptive pill. EHC = emergency hormonal contraception. IUD = intrauterine device. IUS = intrauterine system. LAM = lactational amenorrhoea method. NFP = natural family planning. POP = progestogen-only pill.

**Figure 2.**
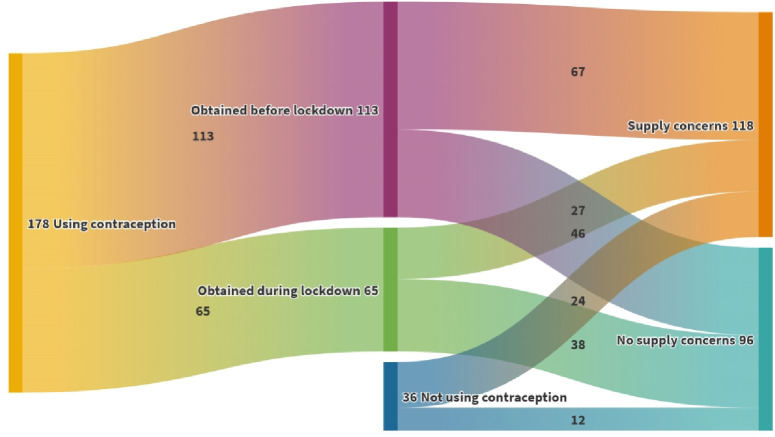
Sankey diagram summarising concerns about contraceptive supplies among the three groups of responders: those using contraception obtained before or during lockdown, and those not using but were seeking a method

Responses were analysed based on three groups of responders as described earlier: those looking to start contraception during lockdown; those who needed a repeat supply or change of methods; and those who obtained contraception during lockdown. Figure 1 summarises contraception methods sought or used by these three groups; the size of each box corresponds to the number of women using or seeking that method. Over half of the responders (55.1%, *n* = 118) anticipated or experienced contraceptive supply problems during the lockdown. Importantly, these women were from all three mutually exclusive groups as shown in Figure 2 (Sankey diagram) and Table 2.

**Table 2.** Summary of responders‘ contraceptive use and needs

Responders grouped by contraceptive use at time of survey	Using contraception method, *n* (%)		Not using contraception method, *n* (%)
	Obtained before lockdown	Obtained during lockdown	Sought during lockdown
**Method used or sought**			
COCP	25 (22.1)	30 (46.2)	8 (22.2)
Male condom	25 (22.1)	10 (15.4)	8 (22.2)
Implant^a^	23 (20.4)	0 (0)	5 (13.9)
IUD^a^	5 (4.4)	1 (1.5)	5 (13.9)
POP	14 (12.4)	17 (26.2)	3 (8.3)
IUS^a^	13 (11.5)	2 (3.1)	2 (5.6)
Depo-Provera^a^	3 (2.7)	4 (6.2)	2 (5.6)
Patch	1 (0.9)	0 (0)	1 (2.8)
NFP	2 (1.8)	0 (0)	1 (2.8)
Sterilisation	0 (0)	0 (0)	1 (2.8)
Sayana Press^a^	1 (0.9)	0 (0)	0 (0)
EHC	1 (0.9)	1 (1.5)	0 (0)
LAM	0 (0)	0 (0)	0 (0)
**Total**	**113** (**100**)	**65** (**100**)	**36** (**100**)
**Source of method**			
General practice	49 (43.4)	34 (52.3)	N/A
CASH clinics	38 (33.6)	6 (9.2)	N/A
Supermarket	11 (9.7)	4 (6.2)	N/A
Online	8 (7.1)	9 (13.8)	N/A
Hospital	3 (2.7)	1 (1.5)	N/A
Abortion clinics	2 (1.8)	1 (1.5)	N/A
Pharmacists	1 (0.9)	10 (15.4)	N/A
Other	1 (0.9)	0 (0)	N/A
**Total**	**113** (**100**)	**65** (**100**)	**N/A**
**Concerns about supply**	67/113 (59.3)	27/65 (41.5)	24/36 (66.7)
**Reason**			
No LARC at clinic or general practice	0 (0)	0 (0)	14 (58.3)
Other reasons (free-text comment)	13 (19.4)	7 (25.9)	5 (20.8)
Not familiar with local services	6 (9.0)	5 (18.5)	2 (8.3)
No remote consult at clinic or general practice	0 (0)	9 (33.3)	2 (8.3)
No contraceptive injection at clinic or general practice	0 (0)	0 (0)	1 (4.2)
No appointments at clinic or general practice	48 (71.6)	6 (22.2)	0 (0)
**Total**	**67** (**100**)	**27** (**100**)	**24** (**100**)

CASH = contraception and sexual health. COCP = combined oral contraceptive pill. EHC = emergency hormonal contraception. IUD = intrauterine device. IUS = intrauterine system. LAM = lactational amenorrhoea method. LARC = long-acting reversible contraceptive. NFP = natural family planning. POP = progestogen-only pill.

a LARC methods.

### Responders looking to start contraception during lockdown

A total of 36 out of 214 responders (16.8%) were not using contraception at the time of the survey and were seeking a contraceptive method (Figure 2 and Table 2). The combined oral contraceptive pill (COCP) and male condoms (22.2%, both *n* = 8) were the most sought methods; 38.9% (*n* = 14) seeking LARC. Two-thirds (*n* = 24) perceived or had difficulties getting their preferred contraceptive methods during lockdown. The reasons given included CASH clinics or general practices were not offering appointments to administer LARC, no option for remote consultations, and not being in their usual area of residence.

### Responders on contraception before lockdown needing a change or repeat prescription

A total of 178 out of 214 responders (83.2%) reported using at least one method of contraception at the time of the survey (Figure 2 and Table 2). The COCP was the most used method (30.9%, *n* = 55), 29.2% (*n* = 52) were using a form of LARC.

The study found 63.5% (*n* = 113) had obtained contraception before the lockdown and were looking for repeat prescription or change of method (Table 2). They obtained their contraception before lockdown from general practices (43.4%, *n* = 49) and CASH clinics (33.6%, *n* =38). Sixty-seven (59.3%) anticipated or reported problems getting further supplies during the lockdown (Figure 2 and Table 2). The reasons given included the following: their general practice or clinic was not offering appointments; not knowing how to arrange a consultation; and other free-text comments included not being able to justify it as an ’emergency’ to use health services during lockdown.

### Responders on contraception obtained during lockdown

The study found 36.5% (*n* = 65) of responders who were using contraception at the time of the survey obtained it during lockdown (Figure 1 and Table 2); these included new or repeat prescriptions. COCP was the most used method (46.2%, *n* = 30), followed by POP (26.2%, *n* = 17), and male condom (15.4%, *n* = 10). Fewer responders reported using LARC during lockdown than before (10.8% versus 39.8%). Over half of this group obtained contraception from general practice (52.3%, *n* = 34); CASH clinics dropped from the most common source of contraception before lockdown to fourth during lockdown (Table 2).

Importantly, even though this group obtained contraception during lockdown, 41.5% (*n* = 27) were uncertain where or how to get their next repeat prescription (Figure 2). The reasons included the following: general practice or clinic was not offering any appointments; general practice or clinic was not offering remote consultations; and other free-text comments included not knowing how to get a check-up (for example, blood pressure) and prescription.

### Free-text analysis

A total of 59 free-text responses to the survey were received; the text is summarised as a word cloud (see Supplementary Figure S6) and they are divided into the following themes: access, acceptability, relevance or appropriateness, and equity.

#### Access

Responders, especially those staying away from their usual residence during lockdown, reported how useful it was that general practices and CASH services offered remote consultations for assessment and mailed or used an Electronic Prescription Service (EPS). This is a paper-free method to send prescriptions to a nominated pharmacist to issue contraceptives.^20^ Just under half of the free-text comments on access were about poor signposting as they did not know who to call or contact to get contraception. One had to travel a longer distance to obtain LARC owing to closure of a nearby clinic.

#### Acceptability

A couple of responders were unhappy about changing from their usual and reliable methods to condoms. Responders who wanted LARC reported clinics were suspended so had to use other less preferred contraceptive methods.

#### Relevance or appropriateness

Getting contraception was challenging for those staying away from their usual residence who were unfamiliar with local services, those with anxiety who were unable to go out, and young people staying with parents during lockdown.

Responders using methods with non-contraceptive benefits to treat medical conditions (for example, COCP for acne and IUS for heavy menstrual bleeding) were unable to access them and that meant their symptoms were prolonged or untreated.

### Equity

A couple of responders felt it was unfair when blood tests and childhood immunisations continued during lockdown but not contraceptive injections or implants. Four reported paying for contraception from online pharmacies; two had to buy their own condoms, which would have been free from CASH clinics before the lockdown.

## Discussion

### Summary

To the authors’ knowledge, this is the first peer-reviewed study of women’s access and experiences of contraceptive care during the first wave of COVID-19 lockdown in the UK. The study found over half of all responders reported perceived or actual difficulties in obtaining contraception during the pandemic; some reported clinics were shut, and remote consultation was not available. Some of those who obtained contraception during the lockdown were not able to get their preferred method or had to use less reliable methods. General practice was an important source of contraception during the first lockdown. Remote consultations and prescriptions sent by mail or electronically to a pharmacy were especially welcome for those staying away from their usual residence during lockdown.

In the early phase of the pandemic, professionals and policymakers reported concerns regarding the impact of COVID-19 on access to sexual and reproductive health care globally in high-, middle-, and low-income countries. The study validated this concern but also gave examples of how access could be maintained and improved.

### Strengths and limitations

Online surveys are subject to selection bias and validity of questionnaires used, especially so during this pandemic.^21^ The findings need to be interpreted with caution as a probability sample that was representative of the UK population was not used; there was underrepresentation of responders from outside London and South East England, as well as those aged ≥35 years. Adolescents aged between 16 and 18 years were overrepresented and might have had different contraceptive needs to other age groups.^22,23^ The use of an online survey might have excluded those who might not have used social media regularly or who had sensory impairment or language difficulties. It is possible the findings underestimated the true extent of the issues.

Despite a small non-probability sample, women’s experiences were captured from four countries of the UK and from a wide age range. The use of descriptive and free-text analysis also offered first-hand insights into the experiences of women getting contraception during the lockdown, including both actual and perceived difficulties with access. The high completion rate reduced the risk of reporting bias and reflected the survey’s relevance for responders and the ease of administration. The findings were also consistent with women’s experiences from surveys conducted in other countries during this pandemic.

### Comparison with existing literature

The findings are consistent with reports from other countries that access to contraception was challenging for women during the COVID-19 pandemic. That some of the responders were unsure how to access contraception, thought clinics were shut, and methods such as LARC were unavailable, were similar to those from an online survey of young people aged 16–24 years in Scotland who also thought they could not justify their reproductive needs as ‘essential’; remote consultations also raised privacy concerns while residing with their parents.^24^ Another study from Australia reported lack of access to methods owing to restrictions, particularly LARC products.^25^ Over half of women surveyed in the US experienced barriers to accessing their preferred contraception method as some facilities were closed; the pandemic also worsened existing inequalities to contraception in a country with no universal access to health care, particularly for women from Black and Hispanic communities and those with lower incomes.^26,27^

### Implications for practice

The findings might have useful contributions to the planning of future pandemics. The impact of poor signposting was found to be not unique to just one or two groups, and perceived problems with access could be a barrier to contraceptive care. A recent study reported higher proportions of unplanned and ambivalent pregnancies conceived during lockdown compared with before lockdown in the UK.^28^ More effective campaigns about access to contraception and support could have prevented some of these unplanned pregnancies; this includes awareness of other venues for advice such as community pharmacies, which has played an important role during lockdown in the US.^29^

Digital access has emerged as an important innovation to meet the challenge of health care during this COVID-19 pandemic; the study findings suggest it is also relevant for reproductive health and access for young people.^30–32^ However, they do not offer solutions to those unable to use digital technology owing to language or sensory barriers, and for LARC access.

Lastly, the lockdown measures produced a situation where women in the postnatal period could not get a contraceptive injection, but their babies were able to get their vaccinations. This illustrated how the pandemic exacerbated inequalities as healthcare assess for some groups were prioritised over women and other vulnerable groups; for example, there was unequal access owing to affordability and those with long distances to travel, and those who were less likely to use digital access.^26^ Mitigation measures also need to be consistent and fair to different population groups to reduce further inequalities.

In conclusion, the study highlighted the need for better information and signposting to obtain free contraception during lockdown restrictions. Contraception, including LARC, should be reframed as an essential service with robust signposting for pandemic planning and beyond.
